# Measuring mental wellbeing in children aged 5 to 12 years: validation of the COMPAS-KIDS and COMPAS-PARENTS mental wellbeing scales in the general community

**DOI:** 10.3389/fpsyt.2025.1611208

**Published:** 2025-09-15

**Authors:** Janine R. Lam, Justine M. Gatt

**Affiliations:** 1 Centre for Wellbeing, Resilience and Recovery, Neuroscience Research Australia, Sydney, NSW, Australia; 2 School of Psychology, University of New South Wales, Sydney, NSW, Australia; 3 The Black Dog Institute, Sydney, NSW, Australia

**Keywords:** mental health, well-being, young people, psychometric testing, reliability, validity, hedonia, eudaimonia

## Abstract

**Introduction:**

The ever-rising incidence of mental health issues in children and adolescents is becoming an issue of global concern. One way to tackle this is to track mental wellbeing during development given the critical role of mental wellbeing within mental health more broadly. Unfortunately, there are limited comprehensive measures of mental wellbeing validated for child self-report, particularly in younger age groups.

**Methods:**

Three versions of the COMPAS-W mental wellbeing scale for adults was transformed for use in children and parents: The COMPAS-KIDS for children aged 5–7 years (21 items), COMPAS-KIDS for children aged 8–12 years (22 items), and the COMPAS-PARENTS for parents to report on their child’s mental wellbeing (22 items). These scales were validated in 99 children aged 5–7 years (*n=*43) and 8–12 years (*n*=56), and their parents.

**Results:**

Internal reliability was demonstrated for the COMPAS-KIDS scale in children aged 5–7 years (*a*=0.664) and 8–12 years (*a*=0.784), and for the COMPAS-PARENTS scale in parents of children aged 5–7 years (*a*=0.819) and 8–12 years (*a*=0.861). Test-retest reliability demonstrated good stability over 4–6 weeks for the COMPAS-KIDS scale in children aged 5–7 years (*r=*0.769) and 8–12 years (*r=*0.910), and for the COMPAS-PARENTS scale in parents of children aged 5–7 years (*r=*0.859) and 8–12 years (*r=*0.901). Criterion validity was established in all age groups, with positive associations found between COMPAS, the PedsQL quality of life scale, the School Wellbeing scale, and negative associations with the CALIS anxiety scale. Correlations between child and parent scores showed some divergence in means, suggesting a reliance on parent report alone is not sufficient to capture child wellbeing.

**Discussion:**

The COMPAS-KIDS and COMPAS-PARENTS scales are reliable and valid measures of mental wellbeing for children aged 5 to 12 years. The collection of surveys from both child and parent are recommended when measuring mental wellbeing. Future studies could consider validation of the scales in independent cohorts that include both clinical and non-clinical cohorts.

## 1 Introduction

The incidence of mental health concerns in children and adolescents is rising (1, 2) with peak age of onset being 14 years (3). Given the integral role of mental wellbeing as a crucial part of mental health, a focus on tracking mental wellbeing over time and boosting it in vulnerable groups could be a critical avenue for prevention. This developmental period provides an opportune time for enriched environments or preventative health interventions given the highly plastic nature of the brain during childhood (4–6). Therefore, in a complementary effort to reducing the risk of mental illness, measuring and building mental wellbeing could be prioritized as a key strategy to support the mental health of children (7).

Mental wellbeing is comprised of two key components: hedonic wellbeing (also referred to as subjective wellbeing) (8) and eudaimonic wellbeing (also referred to as psychological wellbeing) (9–12). Hedonic wellbeing refers to a dimension of human happiness that emphasizes the attainment of pleasure and the avoidance of pain. Rooted in classical hedonistic philosophy, it is typically operationalized through the constructs of subjective wellbeing, which includes three core components: life satisfaction, the presence of positive affect, and the absence of negative affect (13). This approach contrasts with eudaimonic wellbeing, which focuses on meaning, self-realization, and psychological functioning. According to Ryan and Deci (14), hedonic wellbeing is grounded in the pursuit of happiness through pleasurable experiences and the satisfaction of desires. Similarly, Waterman (15) clarified that the hedonic perspective evaluates wellbeing based on personal experiences of pleasure and contentment, making it a more experience-oriented and emotion-driven construct. There are several measures of mental wellbeing that reliably assess both hedonic and eudaimonic wellbeing in both adults and adolescents, including the Warwick Edinburgh Mental Wellbeing Scale (WEMWBS) (16, 17), Mental Health Continuum-Short Form (MHCSF) (9), and the COMPAS-W Wellbeing Scale (18), but are yet to be validated in younger children.

There is a myriad of wellbeing measures developed for younger and older children, but these measures vary in the aspects of wellbeing measured, with often limited psychometric testing (particularly in terms of test-retest reliability), and/or limited specificity in the age group tested. For instance, in older children, a comprehensive measure of wellbeing is the 71-item Middle Years Development Instrument for children aged 10–15 years, with good internal reliability (*a*=0.67-0.95), but variable test-retest reliability over 4 to 6-weeks (*r* ranging from 0.40-0.81) (19). For measures of hedonic wellbeing specifically, there is the 5-item Satisfaction with Life Scale adapted for children aged 9–14 years with good internal reliability (*a*=0.86), but test-retest reliability was not reported (7). The Brief Multidimensional Students’ Life Satisfaction Scale for children aged 11–14 years (20), which includes 5 items to assess satisfaction across family, friends, self, school, and living environment, has good internal reliability (*a*=0.75), but test-retest reliability was again not reported. Another is the 12-item Stirling Children’s Well-being Scale validated in children aged 8–15 years, assessing positive emotional states and positive outlook, with good internal reliability (*a*=0.85) and test-retest reliability over 1 week (*r*=0.75) (21). In terms of eudaimonic wellbeing, the 10-item Psychological Wellbeing and Distress Screener has been shown to demonstrate good confirmatory model fit with good internal reliability (*a*=0.75-0.79), but again test-retest reliability was not reported (22). Another is the 20-item Engagement, Perseverance, Optimism, Connectedness, and Happiness Measure of Adolescent Well-being in children aged 9–18 years, with good internal consistency (*a*=0.72-0.98) and test-retest reliability over 3-weeks (*r*=0.75) (23).

Across all of these measures, none have yet been validated in children younger than 7 years, and of the participants tested, the cohorts often mixed children with adolescents rather than validating the surveys separately in developmentally-appropriate age groups. This approach could have compromised the validity of results as children are at a different developmental stage to adolescents, involving different cognitive and reasoning capacities (24). The average child under the age of 7 years will tend to only think about content in concrete terms (i.e., consider one dimension of a situation at a time and be unable to consider other’s perspectives) (25). In contrast, both concrete and abstract thinking starts to emerge in children over the age of 7 years, where multiple dimensions of a situation can be considered simultaneously, in addition to the understanding that others may not share their perspectives (25). Adolescents, on the other hand, are capable of thinking abstractly, entertaining multiple possibilities, and considering long-term outcomes (25). Consequently, validating a metric across different age groups may result in estimates that mask true scale reliability for children versus adolescents.

Further, if we consider the available measures of wellbeing for younger children, there is a relative scarcity of wellbeing measures that assess both hedonic and eudaimonic wellbeing comprehensively. Of the few self-report measures of wellbeing available in younger children, most tend to target hedonic wellbeing alone using measures of life satisfaction, quality of life, or positive affect. One example is the 14-item Multidimensional Students’ Life Satisfaction Scale based on Diener’s adult Satisfaction with Life Scale for children aged 8–11 years (26), with demonstrated reliability (*a*=0.90-0.92) and test-retest reliability over 2 and 4-week periods (*r*=0.70–0.90). For quality of life, the 23-item Pediatric Quality of Life Inventory (PedsQL) measures quality of life of children aged five years and above, with good internal reliability (*a*=0.86-0.91), but no reports on test-retest reliability (27). Another quality of life measure in younger and older children is the 4-item Very Short Well Being Questionnaire for Children (28) which assesses quality of life at school, home, with peers, and for physical health, with adequate internal reliability for children aged 6–7 years (*a*=0.63) and 9–10 years (*a*=0.66), although test-retest reliability was not reported. Using the same sample, Smees et al. (28) adapted the Positive and Negative Effect Schedule for Children to develop the 10-item Definitional Positive and Negative Effect Schedule for Children for participants as young as 6 years of age, with good internal reliability demonstrated for the positive affect sub-scale for children aged 6–7 years (*a*=0.68) and 9–10 years (*a*=0.77). Again, test-retest reliability was not conducted due to the data being cross-sectional. A measure of wellbeing that combines multiple components of hedonic wellbeing is the 21-item ‘How I Feel About Myself and School’ scale for children aged 7–16 years (29), which assesses interpersonal wellbeing, life satisfaction, competence, and negative emotion. The study reported supportive confirmatory factor model structure of the scale, but neither internal reliability or test-retest reliability analyses were reported. In summary, while there are several measures of wellbeing available in younger children, they are largely limited to evaluating aspects of hedonic wellbeing, with no measures dedicated to the assessment of eudaimonic wellbeing in combination, and psychometric evaluation has mostly been limited to internal reliability.

The purpose of the current study was to validate a mental wellbeing scale that assesses both hedonic and eudaimonic wellbeing in children aged 5–12 years, from their perspective and their parents’. To achieve this, we drew on an existing wellbeing scale (the COMPAS-W) validated in adults (30) and adolescents aged 13–17 years (18), and adapted the items to suit younger children in collaboration with speech pathologists and pediatricians. We will term this new scale ‘COMPAS-KIDS’. A parent version of the scale (termed ‘COMPAS-PARENTS’) was tested in parents to help establish reliability between the two scales. The COMPAS-PARENTS scale is comprised of the same items as the COMPAS-KIDS scale except each item asks the parent to report on their child’s wellbeing. Psychometric assessment of both scales will include reliability analyses for internal consistency, and test-retest reliability over 4–6 weeks. Criterion validity will be assessed by examining associations between the COMPAS scales and other mental health and wellbeing measures using the PedsQL (27) for health-related quality of life, the How I Feel About Myself and School questionnaire (School Wellbeing) for school wellbeing, and with anxiety symptoms using the Child Anxiety Life Interference Scale (CALIS) (31). These analyses will be conducted in children split by age (5–7 and 8–12 years) to encapsulate developmental trends.

## 2 Materials and methods

### 2.1 Ethical standards

Ethical approval was obtained from the SWSLHD Human Research Ethics Committee (2021/ETH00092). Parent consent for themselves and their child was obtained through Qualtrics prior to completing the survey, and child consent was given verbally to the researcher, who then recorded this consent in the Qualtrics form prior to taking the child through the survey.

### 2.2 Participants

The original sample comprised 124 children and their parents residing in Australia or New Zealand. A total of 25 children were excluded due to invalid responses from the parent or child and/or siblings being present in the sample. In addition, only complete parent-child pairs were included in Wave 1 as we required matched data for parent-child analyses. Wave 1 analyses therefore included 99 children and their parents/caregivers from the general public (43 children aged 5–7 years and their parents and 56 children aged 8–12 years and their parents). Of the Wave 1 paired sample, 87 children (38 children aged 5–7 years, 49 children aged 8–12 years) and 84 parents (36 parents of children aged 5–7 years and 48 parents of children aged 8–12 years) also provided Wave 2 data, which was used in test-retest analyses. Our sample size criteria was determined by the most fundamental analyses required for measure validation, internal reliability, complemented by correlation analyses. Evidence from Yurdugül’s (32) study shows a sample size of at least *n*=30 is required for robust estimation.

Inclusion criteria for eligibility into the study included children being aged 5–12 years and both parent and child being able to understand verbal English, willing to give consent and comply with the study, and availability for both baseline and follow-up surveys one month later. Families from the general public were included, and so children with either the presence or absence of psychiatric or neurodevelopmental conditions were included in the study, as well as non-biological primary caregivers. Baseline data was collected between November 2021 and February 2024.

### 2.3 Procedure

To recruit participants, print and digital study advertisements were posted via various sources, including Facebook (paid and organic posts), LinkedIn, Microsoft teams, Instagram, Google AdWords, another children’s research study, volunteering sites, private/independent school and pre-school newsletters, emails to professional networks (Local Health District clinics, Neuroscience Research Australia (NeuRA), Australian university psychology and public health departments), radio, community swimming schools, seminar talks, and parent networks. Each study flyer and digital blurb included a hyperlink to information about the study provided for public viewing on the NeuRA website.

Interested parents were requested to submit their contact details via the NeuRA study page. The research team sent an email to all parents who expressed interest in the project, explaining what the study would involve, eligibility criteria, and their right to not answer questions or stop participating at any time without penalty. Once the parent confirmed interest in the project and eligibility, appointment times were scheduled with the parent and child, for the child to complete the survey by Zoom video call with the research team member. Ahead of the first appointment, parents were also sent a Qualtrics link for them to read the Participant Information Statement and provide online consent for themselves and their child (as their guardian), following which the first parent survey was available for completion.

During the later Zoom call with the child, the research team member introduced themselves, provided a pictorial explanation of what the study was about, and received verbal assent from the child to participate in the study prior to survey completion. Parents were permitted to be present during the child’s interview as long as they did not assist the child with answering the survey questions. To complete the baseline survey battery, the researcher read out each question aloud for the child to answer, the child answered each question verbally, and then the researcher entered the child’s responses into the child’s Qualtrics survey on the researcher’s computer. For each survey, the researcher explained the response options for the child, and that there weren’t any ‘right’ or ‘wrong’ answers before proceeding to ask the child the survey questions. The child was permitted to ask for clarification of the questions asked and to decline answering any questions they didn’t want to answer. Accuracy of responses was ensured through pilot testing and collecting parent-report of the children’s responses. Children were also provided the opportunity to respond to a ‘practice’ question to ensure they understood what was required of them before the researcher proceeded to ask the test questions in the survey battery.

At follow-up (4–6 weeks later), the same process was repeated to collect the child’s Wave two survey data. (We aimed to schedule the follow-up survey 30 days following baseline, however this was not always possible due to parent and/or child availability).

At baseline, all surveys were completed, with survey completion time for children ranging from 6–15 minutes, and up to 30 minutes for parents. At follow-up, only the COMPAS-KIDS, COMPAS-PARENTS, and PedsQL surveys were repeated in children and parents; as such, the survey completion times were reduced to 6–8 minutes for the children, and 15 minutes for the parents. A prize draw to win one of five $100 supermarket vouchers upon completion of the second survey was offered as an incentive for participation.

### 2.4 Measures

The parent and child surveys included measures of demographics (e.g., age, sex, education, socio-economic status), psychiatric medical history, health and lifestyle factors, wellbeing, anxiety/depression symptoms, problem, and prosocial behaviors. Parent surveys included questions about parents and their children. All questions about the child’s family referred to the family they lived with. Each survey response scale provided the option to answer *“I don’t know/Prefer not to answer”*. Scale versions sometimes varied depending on the age of the child.

#### 2.4.1 COMPAS-KIDS Wellbeing Scales (for children aged 5–7 and 8–12 years)

The COMPAS-KIDS Wellbeing Scale measures hedonic and eudaimonic mental wellbeing of children aged 5–12 years. The items and sub-scales were derived and adapted from the 26-item COMPAS-W Wellbeing Scale for adults (30). It was developed from exploratory and confirmatory factor analyses which identified a common factor of total wellbeing and six sub-scale components of Composure, Own-worth, Mastery, Positivity, Achievement, and Satisfaction (30). High internal reliability (*α*=0.840) and test-retest reliability (*r=*0.818) over 12 months was established in 1,669 adults aged 18–61 years (30). The factor structure and reliability was also later established in a cohort of 1,078 adolescents aged 12–17 years, with good internal reliability (*α*=0.880) and test-retest reliability (*r=*0.845) over 4 weeks (18).

For COMPAS-KIDS, we wanted to draw from the items and structure of COMPAS-W to create a scale suitable for children aged 5–12 years. For this, we created two versions to suit the varying cognitive abilities apparent in children aged 5–7 years versus 8–12 years (25). These two versions of the scale varied in two key ways: (1) fewer response options (3-point) for younger children *vs* 5-point options for older children, and (2) item wording variations for which simpler wording or reduced items were used for younger children than older children. These variations were achieved via several phases of development. In the first instance, JMG reviewed each of the items with an 8-year-old child to obtain their understanding of the meaning of each item, and sought alternative simplified wording from the child for their age-related and younger peers. This new set of worded items was then reviewed by several pediatric specialists. Speech pathologists from the South West Sydney Stuttering Unit initially reviewed the new COMPAS-KIDS items for another related study in children who stutter (study in progress). The clinicians confirmed or further simplified the wording of the items for the different age groups. To shorten the scale, they also recommended the removal of two items (items 15 and 26 from the original adults COMPAS-W), which were similar in wording to two items that remained in the scale (items 13 and 25 from the original adults COMPAS-W, respectively). It was also decided to change the Likert response scale for the younger age group (5–7 years) from a 5-point to a 3-point response scale of (1=‘no’, 3=‘sometimes’, 5=‘yes’). Fewer response options were provided in the 5–7 year-old version of COMPAS-KIDS to simplify answering of questions thereby maximizing the chance of collecting accurate responses (33) given younger children’s limited cognitive ability (25). Also, compared with older children, younger children tend to respond more on extreme ends of the scale when self-reporting on affect due to their developmental stage (25, 33), thus requiring a less nuanced scale. Further, the three-option response format was developed in alignment with widely-recognized validated mental health measures for young children in research and practice, such as the Pediatric Quality of Life inventory (27). The scale for children aged 8–12 years retained the original five-point response scale (1=‘strongly disagree’, 2=‘disagree’, 3=‘neutral’, 4=‘agree’, 5=‘strongly agree’).

A second review of the items was then conducted in consultation with an independent group of pediatric specialists (a pediatrician, a speech pathologist, and a child psychiatrist) from South West Sydney Local Health Districts and UNSW. These clinicians recommended further refinements to the items, including the use of simpler terminology, and the provision of examples to some items to aid comprehension. In addition, the clinicians advised the removal of two further items for all children (items 11 and 12 from the original COMPAS-W adult scale) as it was suggested these items assessed behaviors that were not developmentally suited for this age group (i.e., opportunities for decision making and consideration of different perspectives). For 5-7-year-old children, the additional removal of item 16 from the original scale was recommended for similar reasons as it assessed goal setting. Together, these revisions resulted in a 21-item COMPAS-KIDS scale for children aged 5–7 years, and a 22-item COMPAS-KIDS scale for children aged 8–12 years. The same items were used across children aged 5–7 and 8–12 years, but with the addition of item 13 for 8-12-year-old children only (labelled as item 13 in the supplementary material, but labelled as item 16 in the original adult scale), with different response scales across the age groups. The supplementary material lists the final items used in the COMPAS-KIDS scales for the 5–7 and 8-12-year-old age groups, and corresponding response scales. Items are summed to calculate total wellbeing, taking into consideration any reverse-coding, with higher scores indicating higher wellbeing.

#### 2.4.2 COMPAS-PARENTS Wellbeing Scale (for parents of children aged 5–12 years)

The COMPAS-PARENTS Wellbeing Scale comprises the same items as the COMPAS-KIDS scale for 8-12-year children (22 items), and is applied to all children aged 5–12 years. The measure adopts a 5-point scale (1=‘strongly disagree’, 2=‘disagree’, 3=‘neutral’, 4=‘agree’, 5=‘strongly agree’). See supplementary material for corresponding items for COMPAS-PARENTS. Items are summed to calculate total wellbeing, taking into consideration any reverse-coding, with higher scores indicating higher wellbeing.

### 2.5 Criterion validity measures

The following age-appropriate scales were used as criterion measures to validate COMPAS-KIDS and were administered to the parents and/or children.

#### 2.5.1 The Pediatric Quality of Life Inventory: children (5-7, 8–12 years) and parents

The 23-item PedsQL measures health-related quality of life in children from clinical and non-clinical populations (27) and was administered to all children and parents. The measure asks children about problems they have had with their functioning across four domains in the past few weeks: physical functioning (8 items), emotional functioning (5 items), social functioning (5 items), and school functioning (5 items). The parent version of the PedsQL asks for the parent’s assessment of the child’s quality of life using the same items included in the child’s survey. Items are scored on a 3-point response scale for children aged 5–7 years [0 (‘not at all’), 2 (‘sometimes’), and 4 (‘a lot’)], and on a 5-point response scale for children aged 8–12 years and parents [0 (‘never’), 1 (‘almost never’), 2 (‘sometimes’), 3 (‘often’), and 4 (‘almost always’)]. Scoring requires reverse coding and transforming (0=100, 1=75, 2=50, 3=25, 4=0) before calculating total scores. The total mean score (sum of items over number of items across all domains) was used in this study for children and parents. Previous studies show the correlations between parent and child reports using this scale range from 0.42-0.68 (27).

#### 2.5.2 School Wellbeing Questionnaire: children (8–12 years only)

The 21-item School Wellbeing questionnaire assesses a child’s wellbeing within the school context (29). It was originally validated for children aged 7–16 years old, so here it was administered to children aged 8–12 years only. The scale assesses four dimensions: interpersonal wellbeing (8 items), life satisfaction (6 items), perceived competence (5 items), and negative emotion (5 items). A 3-point response scale is used: 1 (‘not often’), 3 (‘sometimes’), 5 (‘often’) and all items are summed for a total score.

#### 2.5.3 The Child Anxiety Life Interference Scale: children (6-7, 8–12 years) and parents

The 9-item CALIS measures the impact of anxiety on a child’s life (31) and has been validated for children 6–17 years old and was therefore administered only to children 6–12 years. The measure first asks the child *“Do fears and worries upset or distress you?”* and then asks how much fears and worries make it difficult for the child for eight different situations at home and outside the home. Items are answered on a 5-point scale: 0 (‘not at all’), 1 (‘only a little’), 2 (‘sometimes’), 3 (‘quite a lot’), and 4 (‘a great deal’). We used the total score (sum of all items) in our analyses. The parent version asks for the parent to report on the same items as the child version. Previous studies show the correlations between parent and child reports using this scale range from 0.37-0.50 for the subscales (total score correlations were not reported) (31).

### 2.6 Pilot testing

We asked the first 10 families (parents and children) recruited to the study for feedback on the wording of the COMPAS-KIDS scales before collecting data from the remaining children and parents. We asked children to identify any COMPAS-KIDS questions they didn’t understand. We also asked parents to identify any COMPAS-PARENTS questions they didn’t understand and to suggest alternative wording to make the questions more meaningful and coherent. By refining the survey with feedback from a small sub-set of families, we hoped to enhance the survey’s ability to accurately measure wellbeing in the majority of participants, thereby saving effort and time on further testing and iterations of the survey.

Feedback from the first 10 children suggested that two COMPAS-KIDS items needed editing. These questions consistently required clarification by children before they could answer them and were therefore simplified for the remaining participants. The use of the word “often” in *“9. Are you often sick?”* (as numbered in the supplementary) was removed and the item was converted to *“Do you feel like you get sick all the time?”.* The use of the word “prepared” in *“11. Do you like to be prepared for new things?”* (as numbered in the supplementary) was removed and the item was converted to *“Do you like to be ready for new things?”.* The parents did not report any consistent issues with the item wording of the COMPAS-PARENTS scale, apart from item 11 which was also changed to *“Does your child like to be ready for new things?”* in the parent scale (as numbered in the supplementary).

### 2.7 Analyses

#### 2.7.1 Data cleaning

During data collection, the interviewer identified several participant responses as invalid and these were subsequently removed from the dataset, *n*=7 (children 5–7 years, *n*=5, and children 8–12 years, *n*=2). Responses were deemed invalid when the interviewer observed that the parents answered questions on behalf of their children in the child’s survey (despite being advised not to do so), or when children had severe attention issues affecting their capacity to answer survey questions. Participant responses were also excluded for the current analyses if the child had a sibling participating in the study, *n=*18 (children 5–7 years, *n*=4 and children 8–12 years, *n*=14). For these cases, we used R to randomly select and include one sibling’s responses (along with their parent’s responses) for analysis, and excluded the responses of the other sibling(s) to prevent familial confounding. For Wave 1, child data was matched to parent data, with one parent and child pair excluded from the analysis as multiple parents answered the one parent survey. Missing data was replaced with the series mean for cases when < 10% of the data was missing. If outliers were identified, they were kept in the dataset to maintain natural variation in the data. The data was checked for normality using Q-Q plots. Where partial ages of children were given by parents we rounded these down to the next whole number.

#### 2.7.2 Reliability and validity

All analyses were conducted separately for parents and children, with the child data further stratified for the 5-7- and 8-12-year age groups in order to reflect developmental changes in the age groups. To assess reliability, internal reliability for COMPAS-KIDS, COMPAS-PARENTS, the PedsQL, School Wellbeing, and CALIS was assessed for total scores using Cronbach’s alpha with recommended thresholds being ≥ 0.70 (34). Test-retest reliability over 4–6 weeks was assessed using intra-class correlation coefficient analyses, with *n*=6 data excluded from test-retest analyses due to data collection being more than six weeks (42 days) since baseline.

To assess validity, criterion correlations were conducted to examine the relationship between COMPAS-KIDS and the other child wellbeing and anxiety scales. For children aged 5–7 years, COMPAS-KIDS was correlated with the PedsQL and the CALIS, whereas in children aged 8–12 years, COMPAS-KIDS was correlated with the PedsQL, the CALIS, and the School Wellbeing questionnaire. Reliability between the parent and child measures was assessed using Pearson correlations (*r*) between the COMPAS-KIDS and COMPAS-PARENTS scores at baseline. (If more than two days had lapsed between a parent and their child’s surveys, the COMPAS-KIDS and COMPAS-PARENTS scores were excluded for that family pair from parent-child correlation analyses, *n*=4 pairs). We performed an independent sample *t*-test to compare the mean scores for children and parents on the COMPAS-KIDS and COMPAS-PARENTS scales, the child PedsQL and parent PedsQL scales, and the child CALIS and parent CALIS scales to assess whether there was consistency in responses across children and parents responding for their children’s scores.

## 3 Results

### 3.1 Descriptive statistics

The sample characteristics of the parents and children are described in **Table 1**, with the information categorized by the age of the child. For the child samples (5–7 *vs* 8–12 years), other than differences in mean age and sex of the sample (with fewer females in the younger age group than the older age group), the groups showed similar distributions in terms of country of birth, absence or presence of psychiatric or neurodevelopmental diagnosis, and relative cognitive ability. Sample distributions were also similar for the parent samples, with the majority of parents being biological mothers, in married/de facto relationships, born in Australia or New Zealand, having completed high school, working full-time, with a total household income between $100-250K, and an absence of psychiatric or neurodevelopmental diagnoses or conditions.

**Table 1:** Descriptive statistics for demographic variables for child and parent participants.

Demographic variables	For children aged 5–7 years *n*=43	For children aged 8–12 years *n=*56
Age, Mean (*SD*)	6.19 (0.82)	9.89 (1.26)
Female, % (*n*)	41.86 (18)	55.36 (31)
Child country of birth, % (*n*)		
Asia	4.65 (2)	7.14 (4)
Africa	4.65 (2)	3.57 (2)
North America	6.98 (3)	5.36 (3)
South America	6.98 (3)	1.79 (1)
Europe	0	1.79 (1)
Australia and New Zealand	76.74 (33)	78.57 (44)
Diagnosis, % (*n*)		
No diagnoses	83.72 (36)	80.36 (45)
Neurodevelopmental condition	11.63 (5)	10.71 (6)
Psychiatric or psychological diagnosis	4.65 (2)	1.79 (1)
Both neurodevelopmental and psychiatric diagnoses	0	7.14 (4)
Relative cognitive ability rating*, *Mean (SD)*	6.88 (1.72)	6.51 (1.8)
	For parents of children 5–7 years *n*=43	For parents of children 8–12 years *n=*56
Age, Mean (*SD*)	39.81 (5.03)	42.77 (5.24)
Female, % (*n*)	90.70 (39)	91.07 (51)
Marital status, % (*n*)		
Single	4.65 (2)	5.35 (3)
In a relationship	0	1.79 (1)
Married/De facto	93.02 (40)	82.14 (46)
Divorced	2.33 (1)	8.93 (5)
Widowed	0	1.79 (1)
Guardian-type, % (*n*)		
Parent	97.67 (42)	98.21 (55)
Adoptive parent/legal guardian	2.33 (1)	1.79 (1)
Parent country of birth, % (*n*)		
Asia	16.28 (7)	25.00 (14)
Africa	4.65 (2)	7.14 (4)
North America	2.33 (1)	5.36 (3)
South America	4.65 (2)	1.79 (1)
Europe	16.28 (7)	12.50 (7)
Australia and New Zealand	55.80 (24)	48.21 (27)
Education, % (*n*)		
Completed high school	97.67 (42)	91.07 (51)
Vocational training	4.65 (2)	16.07 (9)
Bachelor’s degree	34.88 (15)	21.43 (12)
Graduate diploma	13.95 (6)	12.50 (7)
Postgraduate qualification	46.51 (20)	44.64 (25)
Employment, % (*n*)**		
Working full-time	39.53 (17)	48.21 (27)
Working part-time	32.56 (14)	25.00 (14)
Studying or studying and working	18.60 (8)	14.29 (8)
Looking for work	2.33 (1)	0
Not doing paid work and not looking for work	6.98 (3)	7.14 (4)
Caregiving and home duties	0	10.71 (6)
Total household income, % (*n*)		
$0 - $100,000	18.60 (8)	21.43 (12)
$100,001 - $250,000	41.86 (18)	39.29 (22)
Over $250,000	27.91 (12)	21.43 (12)
Prefer not to answer	11.63 (5)	17.85 (10)
Diagnosis, % (*n*)		
No diagnoses	48.84 (21)	53.57 (30)
Neurodevelopmental condition	6.98 (3)	3.57 (2)
Psychiatric or psychological diagnosis	41.86 (18)	28.57 (16)
Both neurodevelopmental and psychiatric diagnoses	2.33 (1)	14.29 (8)

*Relative cognitive ability rating: 10=Very advanced for child’s age, 5=Equally comparable to other children of their age, 1=Child struggles to do many things appropriate for their age. **Parent employment may sum to over 100% as some parents chose multiple options.

### 3.2 Scale means and variability

Scale means, standard deviations (*SD*), and score ranges for the COMPAS-KIDS and COMPAS-PARENTS survey measures are reported in **Table 2**, across children and parents at baseline.

**Table 2:** Means ± SD (range) for all survey measures at baseline.

	Children 5–7 years	Children 8–12 years	Parents of children 5-7	Parents of children 8-12
	*n=*43	*n=*56	*n=*43	*n=*56
	*Mean (SD) Min-Max*	*Mean (SD) Min-Max*	*Mean (SD) Min-Max*	*Mean (SD) Min-Max*
COMPAS-KIDS	85.51 ± 8.90 (63 - 103)	88.46 ± 9.72 (46 - 104)	–	–
COMPAS-PARENTS	–	–	83.51 ± 9.15 (64 - 100)	79.52 ± 11.14 (53 - 106)
PedsQL	74.17 ± 11.47 (43 - 93)	72.72 ± 12.75 (41 - 96)	73.49 ± 11.61 (42 - 92)	71.98 ± 16.38 (28 - 98)
School Wellbeing	–	79.70 ± 8.11 (51 - 93)	–	–
CALIS	4.91 ± 4.06 (0 - 16)	7.61 ± 4.56 (0 - 21)	5.84 ± 5.7 (0 - 29)	8.26 ± 6.00 (0 - 26)

The values presented in the table are true to each scale version: COMPAS-KIDS (5–7 years) comprises 21 items, whilst COMPAS-KIDS (8–12 years) and COMPAS-PARENTS (5–12 years) are comprised of 22 items each. Transformed equivalent mean ± SD for the COMPAS-KIDS (5–7 years) to a 22-item scale (for comparison) is: 89.58 ± 9.33. PedsQL, The Pediatric Quality of Life Inventory; School Wellbeing, How I Feel About Myself and School questionnaire; CALIS, Child Anxiety Life Interference Scale. The School Wellbeing Scale was only administered to 8 - 12-year-old children.

### 3.3 Internal reliability

In children aged 5–7 years, the internal reliability for COMPAS-KIDS was 0.664, comparable to the reliability of the CALIS at 0.701, and the PedsQL at 0.751 in the same age group (see **Table 3**). In children aged 8–12 years, the internal reliability estimates were higher for all scales, ranging from 0.784 for COMPAS-KIDS and 0.743 for CALIS, to 0.858 for PedsQL and 0.852 for the School Wellbeing scale. For the parent scales, the internal reliability estimates were again higher, with internal reliability ranging from 0.819 to 0.861 for COMPAS-PARENTS in reports for children aged 5–7 and 8–12 years, respectively. Similarly, for the parent PedsQL, the estimates ranged from 0.841 to 0.918, and from 0.904 to 0.862 for the CALIS for parent reports for children aged 5–7 and 8–12 years, respectively.

**Table 3:** Internal reliability for all scales (Cronbach’s Alpha, *α*).

	Children 5–7 years *n=*43	Children 8–12 years *n=*56	Parents of children 5–7 *n=*43	Parents of children 8–12 *n=*56
COMPAS-KIDS	0.664	0.784	–	–
COMPAS-PARENTS	–	–	0.819	0.861
PedsQL	0.751	0.858	0.841	0.918
School Wellbeing	–	0.852	–	–
CALIS	0.701	0.743	0.904	0.862

PedsQL, The Pediatric Quality of Life Inventory; School Wellbeing, How I Feel About Myself and School questionnaire; CALIS, Child Anxiety Life Interference Scale. The School Wellbeing scale was only administered to 8-12-year-old children.

### 3.4 Test-retest reliability

For test-retest reliability over 4–6 weeks, only the COMPAS and PedsQL scales were re-administered in both children and parents. In children, test-retest reliability of COMPAS-KIDS was generally more stable in the older 8-12-year-old children (*r*=0.910, *n*=49) than in the younger 5-7-year-old children (*r*=0.769, *n*=36) (see **Table 4**). These stability rates were higher than that found for the PedsQL over the same time period for older (*r*=0.867) and younger children (*r*=0.737). In parents, the test-retest reliability estimates for COMPAS-PARENTS were comparable across parents of both younger children (*r*=0.859, *n*=33) and older children (*r*=0.901, *n*=47), and again these rates were higher than rates found for the PedsQL in parents of younger (*r*=0.669) and older children (*r*=0.816).

**Table 4:** Test-retest reliability for all scales over 4–6 weeks (intra-class coefficients, ICC).

	Children 5–7 years *n=*36	Children 8–12 years *n=*49	Parents of children 5–7 *n=*33	Parents of children 8–12 *n=*47
COMPAS-KIDS	0.769	0.910	–	–
COMPAS-PARENTS	–	–	0.859	0.901
PedsQL	0.737	0.867	0.669	0.816

PedsQL, The Pediatric Quality of Life Inventory.

To assess criterion validity, the COMPAS-KIDS and COMPAS-PARENTS scales were correlated with the PedsQL, School Wellbeing, and CALIS scales in each child and parent sub-sample (where relevant). The PedsQL correlated positively with COMPAS-KIDS in both child samples, and with COMPAS-PARENTS in both parent samples (see **Table 5**). The School Wellbeing scale also correlated positively with COMPAS-KIDS in the older child sample (it was not collected in the younger child or parent samples). The CALIS anxiety scale correlated negatively with both COMPAS-KIDS and COMPAS-PARENTS in all samples, although the correlation did not reach significance in the younger age cohort (potentially due to the reduced sample size of 32 children, as the scale is only validated for use in children aged 6 years and above).

**Table 5:** Correlations between the COMPAS-KIDS, COMPAS-PARENTS, and other criterion scales.

	COMPAS-KIDS children 5–7 years *n=*43	COMPAS-KIDS children 8–12 years *n=*56	COMPAS-PARENTS for children 5–7 *n=*43	COMPAS-PARENTS for children 8–12 *n=*56
PedsQL	0.614**	0.633**	0.510**	0.656**
School Wellbeing	–	0.643**	–	–
CALIS	-0.098^Φ^	-0.599**	-0.357*	-0.575**

PedsQL, The Pediatric Quality of Life Inventory; School Wellbeing, How I Feel About Myself and School questionnaire; CALIS, Child Anxiety Life Interference Scale. The School Wellbeing scale was only administered to 8-12-year-old children. ^Φ^In the younger child sample, CALIS was only collected in 6-7-year-old children (not 5-year-old children), so *n* was reduced to 32 for this correlation. *p-value = 0.019. **p-value < 0.001.

### 3.5 Parent-child scale reliability

To assess whether the child and parent versions of each scale were measuring similar constructs, we correlated them together for the COMPAS, PedsQL, and CALIS measures. For this analysis, we excluded 2 parent-child pairs from the 5-7-year-old sample as the child surveys were completed more than 2 days (10–21 days) after the parent surveys. In the younger age group (5–7 years), none of the child and parent scales correlated significantly with each other for the COMPAS, PedsQL or CALIS measures (see **Table 6**). In the older age group (8–12 years), the COMPAS-KIDS and COMPAS-PARENTS scales again did not correlate with each other; however, the PedsQL and CALIS measures did correlate positively.

**Table 6:** Correlations between child and parent reports of the same scales.

	5–7 years			8–12 years		
	*r*	*p*-value	*n*	*r*	*p*-value	*n*
COMPAS-KIDS & COMPAS-PARENTS	0.037	0.819	41	0.027	0.847	54
PedsQL (child) & PedsQL (parent)	0.148	0.355	41	0.271	0.047	54
CALIS (child)* & CALIS (parent)	-0.187	0.305	32	0.281	0.040	54

PedsQL, Pediatric Quality of Life Inventory; CALIS, Child Anxiety Life Interference Scale, *CALIS tested in both age groups, but given it has only been validated in children above the age of 6 years, it was only administered to 6-7-year-old children, hence the sample size is reduced to *n* = 32.

To assess whether the mean scores were comparable between child and parent versions, the scores for COMPAS-KIDS (5-7) 21-item version were adjusted to the 22-item scale range versions of the COMPAS-KIDS (8-12) and COMPAS-PARENTS scale, and compared using *t*-test comparisons. COMPAS-KIDS and COMPAS-PARENTS did differ on total means in both age groups, for which children rated their own wellbeing higher on average than their parents for both the younger age group (child 5–7 years, *mean*=89.584, *SD*=9.327, parent *mean*=83.512, *SD*=9.151, *t*-value=3.047, *df*=84, *p*=0.003) and older age group (child 8–12 years, *mean*=88.456, *SD*=9.717, parent *mean*=79.522, *SD*=11.137, *t*-value=4.523, *df*=110, *p* < 0.001). In contrast, for both the PedsQL and CALIS scales, the child and parent mean scores did not differ significantly from each other, and this was evident when comparing both younger or older children subsets (*p* > 0.05). These mean differences in COMPAS scores may partly account for the non-significant parent-child correlations reported for the COMPAS scales in **Table 6**. Yet, they cannot account for the lack of significant parent-child correlations for the PedsQL and CALIS evident in the younger sample, which may be due to inter-item variability.

## 4 Discussion

Childhood is a critical period of physical and neural development, and also a time when mental health issues can first arise. High brain plasticity during this phase also provides a valuable opportunity to intervene and even facilitate states of flourishing. Unfortunately, few comprehensive self-report mental wellbeing measures exist for children, particularly younger children. Our study therefore aimed to examine whether children aged 5–7 years and 8–12 years were able to report on their own mental wellbeing using COMPAS-KIDS. These outcomes were validated against reports of their wellbeing from the children’s parents using COMPAS-PARENTS and other criterion measures.

### 4.1 Measuring mental wellbeing in 5-7-year-old children using self-report

In younger children, reliability and validity estimates supported the utility of the COMPAS-KIDS scale to assess wellbeing in this age group. Although internal reliability for COMPAS-KIDS was on the borderline of being acceptable in younger children (0.664), test-retest results showing the measure to be stable over time and the negative correlation with the CALIS indicated the measure to be reliable and valid for younger children. While the internal reliability estimate was not as high as the other scales, they were within a similar vicinity of values: CALIS (0.701) and PedsQL (0.751). Similar estimates were previously reported for the Very Short Well Being Questionnaire for Children which assesses overall wellbeing in 6-7-year-old children, with internal reliability estimates of 0.630. Given the Very Short Well Being Questionnaire for Children is a much shorter and simpler scale and was tested on a much larger sample (*n*=702), the COMPAS-KIDS performed quite well in comparison for the current sample of 43 5-7-year-old children. Nonetheless, to improve these estimates further, future iterations of the COMPAS-KIDS scale could consider providing more examples to support comprehension of the items. For instance, the researcher who collected data for the current study observed that a few children required further explanation of some questions before being able to answer them. These questions included: *“7. Do you sometimes want to hide because of something you have done?”*, “*11. Do you like to be ready for new things?”*, and *“22. Are you proud of yourself when you do something well, even if your teacher or mum/dad don’t see what you have done?”* (as numbered in the supplementary material). Given younger children typically think in concrete terms, this cohort may have struggled to comprehend the abstract concepts in these items. Test-retest reliability over 4–6 weeks did however suggest that COMPAS-KIDS was stable in this age group (0.769) and slightly more stable than the PedsQL (0.737). Validity estimates were also positive and supportive when comparing COMPAS-KIDS against the quality of life PedsQL scale. A negative correlation was evident with the CALIS anxiety scale as expected, although this association did not reach significance in the younger age group, which may have been due to a reduced sample size for this specific correlation as the CALIS is only validated in children aged 6 years and above. Together, the results suggest COMPAS-KIDS provide children as young as 5–7 years old a unique measure of both hedonic and eudaimonic wellbeing. However, we note internal reliability was on the borderline of being acceptable and could be improved through further refinement of the survey items in future iterations.

### 4.2 Measuring mental wellbeing in 8-12-year-old children using self-report

The reliability and validity values for COMPAS-KIDS in the older children were stronger overall, in comparison to the younger sample. Internal reliability for COMPAS-KIDS was good in older children (0.784), and superior to the CALIS (0.743). While COMPAS-KIDS did not demonstrate as high reliability as the PedsQL (0.858) and School Wellbeing scale (0.852) in the same age group, this may be at the added benefit of having a scale that measures both hedonic and eudaimonic components rather than a single dimension of functioning. Interestingly, the researcher involved in data collection noted that a few children in the 8-12-year-old sample struggled with the some of the same items that the 5-7-year-old sample found challenging (i.e., items 7, 11, 22, supplementary material), and a couple of children also struggled to comprehend some questions many 5-7-year-old children did not, e.g., *“1. Are you happy with your health?”*. This variability in comprehension reflects some diversity in development of abstract thinking within age groups that may be emerging in older age groups, even if the majority of 8-12-year-old participants were able to answer these questions. Tomyn and Cummins also noted variability in responses to domain-based measures of hedonic wellbeing in children under the age of 12 years due to differing cognitive abilities (35). This presents a challenge to developing wellbeing measures for children, even when separated by age group. Nonetheless, COMPAS-KIDS was a reliable measure of wellbeing in our cohort, and the other psychometric results supported its reliability and validity. Test-retest reliability over 4–6 weeks for COMPAS-KIDS was very high in the older children (0.910), and superseded the stability of the PedsQL (0.867). COMPAS-KIDS also demonstrated strong positive relationships with both the PedsQL and School Wellbeing scale, and a strong negative relationship with the CALIS, as expected.

### 4.3 Measuring mental wellbeing of children using parent report

Measuring child wellbeing using parent report appeared to be valid when considering reliability and validity estimates within parents, yet appeared to unravel when correlations between parent and child reports and mean scores were considered. Internal reliability of COMPAS-PARENTS was strong when reporting on younger (0.819) or older children (0.861), as was test-retest reliability over 4–6 weeks for both age groups (0.859 and 0.901, respectively). These internal reliability estimates were on par, or in similar ranges to, internal reliability estimates for the PedsQL (0.841 and 0.918, respectively) and CALIS (0.904 and 0.862, respectively) reported by parents for both age groups, and superior in terms of stability over time as compared to the PedsQL for both age groups (0.669 and 0.816, respectively). Criterion validity was also supportive with strong positive relationships evident with the PedsQL and negative relationships with the CALIS for COMPAS-PARENTS across both age groups.

What was nonetheless unexpected, was the non-significant correlations between the parent scales and the child scales across the COMPAS, PedsQL, and CALIS measures in the younger children, and the non-significant correlations between the parent scales and the child scales for the COMPAS in the older children. This suggests that parents and children are mostly not consistent in their scoring of the child’s behavior, and this is across mental wellbeing, quality of life, and anxiety–particularly in the younger age group of 5-7-year-old children. In fact, for anxiety, the correlation was negative, suggesting opposing reports of the same symptoms which is concerning if these reports are used in clinical practice. In contrast, there was some (albeit weak) alignment between parent and child scores for the PedsQL and CALIS in the older children (8–12 years), but again not for wellbeing. When we compared the mean scores for wellbeing across children and parents, the children always reported higher scores than the parents, across both age groups. These results suggest reliance on parent report alone could be misleading and not representative of the child’s feelings and thoughts about their own mental health and wellbeing, particularly in younger age groups. Alternatively, demand characteristics may have cued children to inflate their survey responses to meet their perceived expectations from nearby adults through minimizing negative and maximizing positive affect and behavior, thus scoring higher in wellbeing on average. Qualitative comments support this contention. We found that when parents listened to their child provide survey responses to the researcher, some parents did comment that they did not expect their child to answer in the way that they did, or they learnt something new about their child from their response. For instance, some parents reported that participation in the study itself sparked conversations with their child about mental wellbeing for the first time. This emphasizes the importance of obtaining children’s self-report of their mental wellbeing rather than relying on parent report alone. Therefore, although this study suggests COMPAS-PARENTS is a more reliable and valid measure of children’s wellbeing than COMPAS-KIDS (particularly for 5-7-year-old children), parent report cannot necessarily provide a proxy of children’s self-report of wellbeing. A child’s own perspective needs to be considered when assessing their wellbeing and making decisions around their care and treatment.

### 4.4 Strengths and limitations

There are several strengths to this study worth highlighting. First, COMPAS-KIDS and COMPAS-PARENTS provide a comprehensive measure of wellbeing for children across both hedonic and eudaimonic wellbeing. Although a few measures of wellbeing (27, 28, 35) have previously been validated for younger children, they focus heavily on assessing hedonic wellbeing alone. Second, COMPAS-KIDS was adapted and validated specifically for children aged 5–7 years and 8–12 years. Cognition during childhood and adolescence varies significantly and requires a deliberate focused approach for measurement. We therefore consulted pediatric experts to cater the language of the items to children of different ages to support comprehension. To then validate the COMPAS-KIDS scales, we used other pediatric mental health and wellbeing scales as criterion measures, which each showed substantial correlations with COMPAS-KIDS. In contrast, the majority of previous studies have validated wellbeing scales in samples that combine children with adolescents (21, 29, 36, 37), without comparing validity within each age group, potentially masking the true reliability and validity of these scales. Another strength of this study is that we recruited from a broad general public distribution, with children and parents from a diversity of cultural backgrounds. Specifically, of the child participants, while 75-80% of the sample were born in Australia and New Zealand, 20-25% were born in a range of other continents including Asia, Africa, North and South America and Europe. Further, of the parents, while about 50% were born in Australia and New Zealand, the remainder were largely from the same countries listed above. The COMPAS-KIDS has therefore been validated in families with large cultural diversity taken into consideration. In contrast, other study samples have typically drawn samples from schools (28) or disadvantaged populations (38), possibly producing a bias in distributions, and/or limiting the external validity of findings. For instance, participants from a school may be influenced by their specific school environment, or come from a particular socio-economic or cultural background. Similarly, children from disadvantaged backgrounds will have particular challenges impacting their wellbeing that may not be experienced by children from non-disadvantaged samples.

Despite the strengths of this study, there are a few limitations worth noting for future developments. First, recruitment of child samples is a difficult process when recruiting through the general population (rather than schools), and as such, our sample size was limited by this difficulty. Future studies with sample sizes of 200 or greater (39) could explore confirmatory factor models by specific age groups to determine varying factor structure with development, or to determine normalized category cut-off scores for clinical and non-clinical groups. Second, we found that some children required adapted wording of the items that referred to school contexts because they either had not yet started school or were home schooled. Therefore, future scale adaptations need to take these alterations into consideration, particularly if the scale is to be used by children without assistance. One other factor to explore is the role of parent sex, which may have influenced parent-child correlations in child wellbeing. As the majority of parent participants were female, future research could consider how the perception of fathers may impact parent-child results, particularly if fathers perceive their child’s wellbeing differently to mothers.

## 5 Conclusion

The COMPAS-KIDS and COMPAS-PARENTS scales are reliable, stable, and valid measures of mental wellbeing that can be used in children aged 5 to 12 years, and their parents. Uniquely, the scale measures both hedonic and eudaimonic wellbeing, providing a comprehensive assessment. The study also identified a divergence in responses when comparing child to parent reports, suggesting that the reliance on parent report alone is not sufficient and needs to be supplemented by child self-report.

## Data Availability

The datasets presented in this article are not readily available because of ethical requirements. Requests to access the datasets should be directed to JG, j.gatt@neura.edu.au.

## Author’s note

JL recruited the participants, collected the data, conducted the statistical analysis and drafted the first version of the paper. JG designed the original COMPAS-W scale and created the adapted versions for COMPAS-KIDS and COMPAS-PARENTS. JG also guided analysis of the data, supervised study coordination and led the broader research program, and provided revisions to all drafts of the paper.
