# Correction: Measuring mental wellbeing in children aged 5 to 12 years: validation of the COMPAS-KIDS and COMPAS-PARENTS mental wellbeing scales in the general community

**DOI:** 10.3389/fpsyt.2026.1876641

**Published:** 2026-06-05

**Authors:** Janine R. Lam, Justine M. Gatt

**Affiliations:** 1 Centre for Wellbeing, Resilience and Recovery, Neuroscience Research Australia, Sydney, NSW, Australia; 2 School of Psychology, University of New South Wales, Sydney, NSW, Australia; 3 The Black Dog Institute, Sydney, NSW, Australia

**Keywords:** mental health, well-being, young people, psychometric testing, reliability, validity, hedonia, eudaimonia

The **Supplementary Material** contained the COMPAS-KIDS and COMPAS-PARENTS scale items. This proprietary instrument was not intended for unrestricted use and has been removed from the Supplementary Material. Further inquiries can be directed to the corresponding author.

The original published findings are unaffected by this amendment.

The file has now been removed.

Since original publication, the intellectual property rights to the COMPAS-KIDS and COMPAS-PARENTS scales have been assigned to Tilt Your Life Pty Ltd, of which Dr Justine Gatt is Founder and Director. The **Conflict of Interest** statement has therefore been updated to read:

"The intellectual property rights to the COMPAS-KIDS and COMPAS-PARENTS scales are assigned to Tilt Your Life Pty Ltd, of which JG is Founder and Director. The scale is available for non-commercial academic and research use and is licensed for commercial use via Tilt Your Life Pty Ltd (contact: justine.gatt@gmail.com)."

The original version of this article has been updated.

